# Effect of the duration of visual exposure to the male on the reproductive performance of nulliparous guinea pigs

**DOI:** 10.29374/2527-2179.bjvm003926

**Published:** 2026-08-12

**Authors:** Hilario Noberto Pujada Abad, Edinson Collas Ostos, Roberto Carlos Tamayo Diaz, Rufino Máximo Maguiña Maza, Víctor Joselito Linares Cabrera, Julio César Valencia Bardales, Miguel Edmundo Lucho-Cerga, Javier Hernán Luyo Flores, Felix Esteban Airahuacho-Bautista

**Affiliations:** 1 Universidad Nacional José Faustino Sánchez Carrión, Huacho, Lima, Peru.; 2 Instituto de Educação Superior Público Huando, Huaral, Lima, Peru.

**Keywords:** estrus, sexual behavior, puberty, Cavia porcellus, non-productive days, estro, comportamento sexual, puberdade, Cavia porcellus, dias não produtivos

## Abstract

With the aim of determining the optimal duration of visual male exposure on the reproductive performance of nulliparous guinea pigs (*Cavia porcellus*), this study evaluated key reproductive indicators from mating to parturition. Sixty 60-day-old females were distributed into three treatments: traditional continuous mating (T_0_), controlled mating with 14-day prior visual male exposure (T_1_), and controlled mating with 7-day prior visual male exposure (T_2_). Results showed that T_1_ significantly increased the frequency of first estrus between days 6 and 10 (67%; p = .02) compared to T_0_ and T_2_ (38%). Additionally, T_1_ showed the highest frequency of afternoon estrus (42%; p < .05) and a higher rate of estrus repetition (p < .05). Regarding reproductive efficiency, T_0_ and T_2_ females exhibited significantly fewer non-productive days (10.5 and 11.3 d, respectively) compared to T_1_ (17.0 d; p < .01). Conversely, the feed cost per female was lowest in T_1_ (7.23 PEN), followed by T_2_ (9.27 PEN) and T_0_ (10.05 PEN; p < .01). No significant differences were observed in litter size (p = .08) or litter weight (p = .23). In conclusion, while 14 days of visual biostimulation prior to mating optimizes estrus synchronization but increases reproductive failures, a 7-day exposure successfully reduces non-productive days and feed expenditure in nulliparous guinea pigs, improving the economic merit of the intensive system without compromising productive parameters.

## Resumo

Com o objetivo de determinar a duração ideal da exposição visual ao macho sobre o desempenho reprodutivo de porquinhos-da-índia (*Cavia porcellus*) nulíparos, este estudo avaliou os principais indicadores reprodutivos desde o acasalamento até o parto. Sessenta fêmeas com 60 dias de idade foram distribuídas em três tratamentos: acasalamento contínuo tradicional (T0), acasalamento controlado com 14 dias de exposição visual prévia ao macho (T1) e acasalamento controlado com 7 dias de exposição visual prévia ao macho (T2). Os resultados demonstraram que o T1 aumentou significativamente a frequência do primeiro estro entre os dias 6 e 10 (67%; p = 0,02) em comparação com T0 e T2 (38%). Além disso, o T1 apresentou a maior frequência de estro no período da tarde (42%; p < 0,05) e maior taxa de repetição de estro (p < 0,05). Em relação à eficiência reprodutiva, as fêmeas dos grupos T0 e T2 apresentaram significativamente menos dias não produtivos (10,5 e 11,3 dias, respectivamente) em comparação ao T1 (17,0 dias; p < 0,01). Por outro lado, o custo da alimentação por fêmea foi menor no T1 (7,23 PEN), seguido por T2 (9,27 PEN) e T0 (10,05 PEN; p < 0,01). Não foram observadas diferenças significativas no tamanho da leitegada (p = 0,08) nem no peso da leitegada (p = 0,23). Conclui-se que, embora 14 dias de bioestimulação visual antes do acasalamento promovam melhor sincronização do estro, essa estratégia aumenta as falhas reprodutivas. Em contrapartida, a exposição visual ao macho por 7 dias reduz os dias não produtivos e os custos com alimentação em fêmeas nulíparas, melhorando o mérito econômico do sistema intensivo sem comprometer os parâmetros produtivos.

## Introduction

In guinea pig (*Cavia porcellus*) production systems, continuous mating is the conventional reproductive strategy, based on permanent male cohabitation to capitalize on postpartum estrus—a highly fertile event (60–80%) occurring 2 to 10 hours after parturition (Harkness et al., 2002). However, continuous exposure to sexual stimuli can lead to satiety or sexual exhaustion of the sire, as males typically show a marked decrease in interest after ejaculation (Young, 1969). Alternatively, controlled mating delays male introduction until several days postpartum or after weaning, allowing rest periods but reducing the annual kidding index (Harkness et al., 2002). Nevertheless, drastic variations in social management and mating frequency can compromise sexual vigor and ejaculate quality, which could correlate with a higher incidence of repeated estrus due to conception failures (Shomer et al., 2015).

To mitigate these restrictions, sociosexual biostimulation, known as the “male effect,” constitutes an effective strategy widely validated in various domestic species to optimize reproductive efficiency (Landaeta-Hernández et al., 2023). This phenomenon relies on the perception of olfactory (pheromones), auditory, and visual stimuli from sexually active males, which activate the hypothalamic-pituitary-gonadal axis (Gelez & Fabre-Nys, 2004). These sensory signals stimulate the pulsatile secretion of gonadotropin-releasing hormone (GnRH) and, consequently, luteinizing hormone (LH), thereby inducing puberty or triggering ovulation in anestrous females (Perry, 2016). Although these neuroendocrine principles are predominantly documented in livestock ruminants, their extrapolation to precocial rodents is biologically plausible. Given that the guinea pig is a spontaneous ovulator sharing homologous neuroendocrine pathways and a highly receptive sensory system, visual male exposure could modulate reproductive performance in nulliparous females (Araníbar & Echevarría, 2014), optimizing intensive production under an animal welfare framework.

In this species, the onset of sexual maturity features remarkable plasticity; although puberty is typically reported around 60 days of age, the presence of adult males can accelerate this process, reducing the age at first estrus to 26 days (Trillmich et al., 2006). This reproductive milestone is defined by the first ovulation and characterized clinically by vulvar congestion, vaginal membrane rupture, and lordosis response (Shomer et al., 2015). Because estrus in guinea pigs is short-lived (8 to 11 hours) and occurs predominantly during the night (Hargaden & Singer, 2012), the integration of continuous video monitoring systems has become essential to guarantee uninterrupted and precise behavioral tracking (Tzanidakis et al., 2021). Under this premise, this research utilized video recording systems to document sociosexual interactions in nulliparous females under different visual male exposure schemes, aiming to evaluate its impact on overall reproductive performance.

## Materials and methods

**Figure 1:**
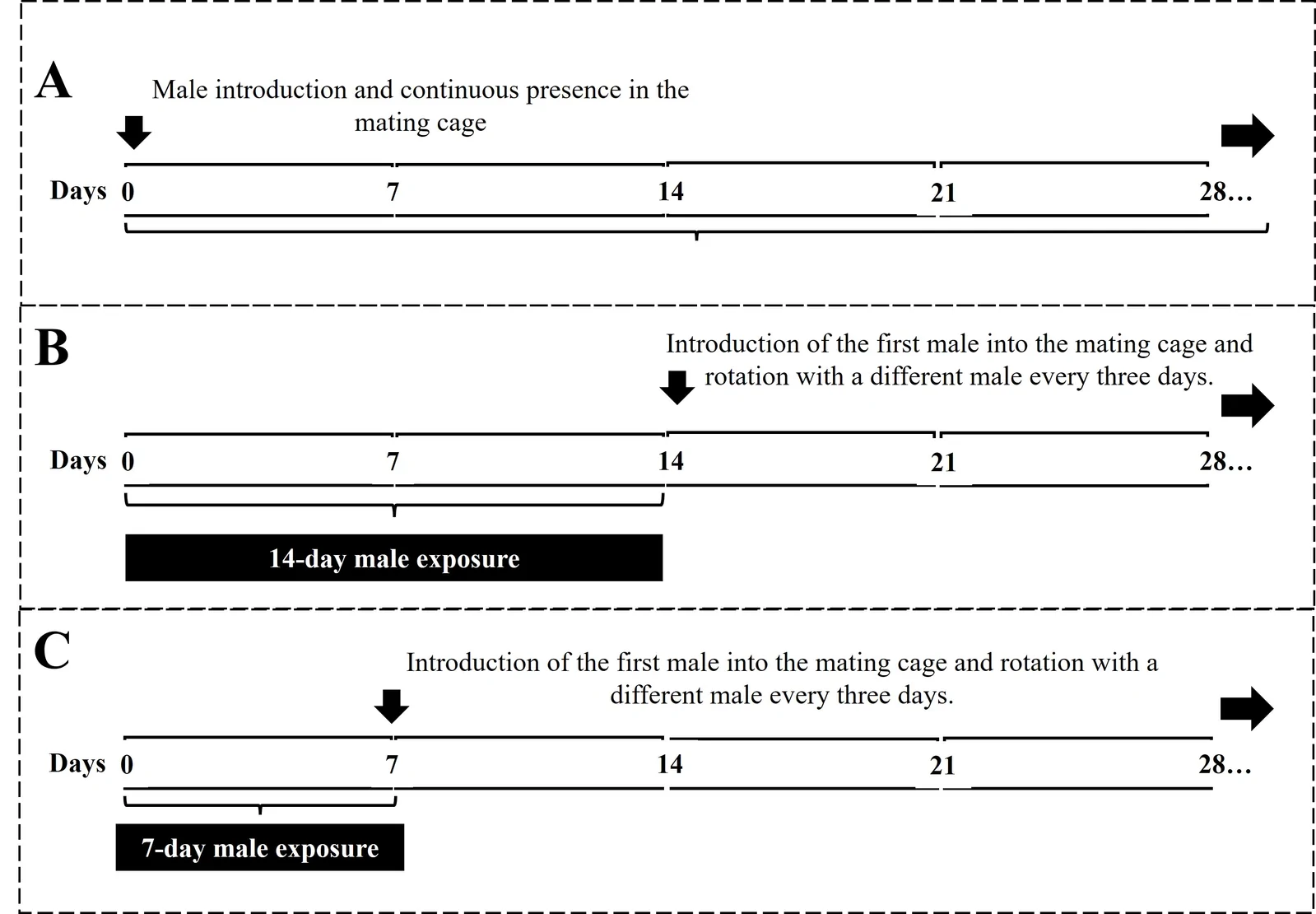
. Timeline of traditional continuous mating (A), controlled continuous mating with 14-day prior male exposure (B), and controlled continuous mating with 7-day prior male exposure (C).

The research was conducted at an experimental guinea pig farm located in the city of Huaura, Peru, at an altitude of 72 m a.s.l. (geographic coordinates: 11°04′12″ S latitude and 77°35′57″ W longitude). Within a climate-controlled facility, nine mating cages (1.42 m^2^ each) were installed, along with four 0.16 m^2^ cages for housing the adult males used for visual exposure. A total of sixty 60-day-old nulliparous females (initial body weight: 695 ± 65 g) were randomly allocated into the nine mating cages, establishing three replicate cages per treatment (n = 3 cages per treatment, with 8 females per cage). To record sociosexual interactions, a video recording system (DVR 4CH Pentabrid 4 MP) was implemented, consisting of three cameras positioned at a height of 2.5 m and strategically distributed to eliminate blind spots, all connected to a computerized control center. The reproductive behavior of the females was continuously monitored from the onset of mating until parturition. Three mating techniques or treatments were evaluated:

1. Traditional continuous mating (T_0_): three replicate cages (8 females and one 90-day-old male placed together per cage) throughout the entire experimental period (from mating to parturition).
2. Controlled mating with 14-day prior male exposure (T_1_): three replicate cages where females were housed without the physical presence of the male but with visual exposure to four cages containing adult males. After the 14-day exposure period, a male was introduced into the cage and rotated every three days with a different male.
3. Controlled mating with 7-day prior male exposure (T_2_): conducted under the same visual exposure scheme, with the male introduced into the cage after 7 days and subsequently rotated every three days (Figure 1).

The diet provided was in pellet form with a nutritional value of 2.9 Mcal/kg of digestible energy, 90% dry matter, 19% crude protein, 8% crude fiber, 1% calcium, 0.8% phosphorus, and 0.2% sodium, supplemented with 35 mg of vitamin C per 100 g of feed. Water was provided *ad libitum* throughout the experimental period.

Reproductive and behavioral variables were assessed through observational analysis of continuous video recordings from the onset of mating until parturition. These included estrus presentation and timing (based on female standing heat), estrus repetition (females returning to estrus after the first service), and number of copulations (defined as effective mating followed by genital grooming by both animals). Furthermore, parturition timing, gestation length (copulation-to-parturition interval), and non-productive days (NPD; the period from the first mating to conception) were recorded. Additionally, the production cost per live-born pup was determined by integrating the days of the reproductive cycle (gestation plus non-productive days), feed intake, and diet cost, divided by the number of live-born offspring.

Qualitative mating indicators were analyzed using Pearson's χ2 test of proportions. To ensure statistical rigor and account for the group-housing conditions, a dual-level approach was implemented for the experimental units. For individual behavioral and qualitative metrics—specifically estrus presentation, timing, and recurrence—the individual female was considered the experimental unit (n = 72). Conversely, for quantitative productive and economic parameters, including litter size at birth, litter weight, feed intake, and production costs, the mating cage was defined as the experimental unit (n = 9 cages; 3 replicates per treatment, with 8 females per cage), and data were aggregated as cage-level means. Continuous variables (litter size and litter weight) were subjected to analysis of variance (ANOVA), following verification of normality and homoscedasticity of the residuals via Shapiro-Wilk and Levene tests, respectively (p > .05). Potential confounding cage effects on individual-level measurements were structurally mitigated by maintaining strictly standardized and identical environmental, nutritional, and stocking density conditions across all replicate cages. All statistical analyses and figures were performed using the R Core Team (2025) statistical software.

## Results

Table 1 presents the estrus indicators in 60-day-old nulliparous females. The frequency of estrus during the first five days after the onset of mating was similar across the three treatments (p = .84). However, between days 6 and 10, females subjected to controlled mating with prior 14-day male exposure (T_1_) recorded the highest frequency (p = .02). Consequently, during the period between days 11 and 15, this same group (T_1_) showed the lowest estrus frequency (p < .05).

**Table 1:** Indicators of estrus presentation in 60-day-old nulliparous females.

	**T_0_ (Traditional)**	**T_1_ (Controlled 14-d)**	**T_2_ (Controlled 7-d)**	**p-value**
**Frequency of first estrus presentation, %**				
0 to 5 days	29	25	25	.84
6 to 10 days	38 *^b^*	67 *^a^*	38 *^b^*	.02
11 to 15 days	33 *^a^*	8 *^b^*	38 *^a^*	< .05
**Time of estrus presentation, %**				
Late night/Early morning (0 to 6 h)	33	33	29	.87
Morning (6 to 12 h)	17 *^a^*	0 *^b^*	29 *^a^*	< .05
Afternoon (12 to 18 h)	21 ^b^	42 ^a^	9 ^c^	< .05
Night (18 to 24 h)	29	25	33	.63
**Estrus recurrence, %**				
Single estrus	100	100	100	
Two estruses (repetition)	17 *^a^*	46 *^b^*	25 *^a^*	<.05
Three estruses (repetition)	0 *^a^*	12 *^b^*	4 *^ab^*	<.05

**Note:** T_0_: Traditional mating scheme (continuous cohabitation with the male); T_1_: Controlled mating with 14-day visual male exposure prior to mating; T_2_: Controlled mating with 7-day visual male exposure prior to mating. Value of *p* determined by Pearson's chi-square (χ2) test of proportions. *^a,b,c^* Different superscripts within the same row indicate statistically significant differences (p < .05). Values without superscripts indicate no significant difference (p > .05).

Regarding the timing of estrus, the presentation was similarly distributed during the late night and early morning (0:00 to 6:00 h) across all treatments. However, during the morning hours (6:00 to 12:00 h), females in the continuous (T_0_) and 7-day exposure (T_2_) groups showed a significantly higher frequency (p < .05), whereas the highest frequency during the afternoon (12:00 to 18:00 h) was observed in the 14-day exposure group (T_1_) (p < .05). Additionally, T_1_ exhibited a significantly higher rate of estrus repetition (two or three cycles) compared to the other groups (p < .05).

Mating indicators for the 60-day-old nulliparous females are shown in Table 2. A trend toward a higher frequency of 0 to 5 copulations was observed in controlled mating females (p = .07), while a trend toward a higher frequency of 6 to 10 copulations was noted in continuous mating (T_0_) and 7-day exposure (T_2_) females (p = .06). Furthermore, the highest frequency of 11 to 20 copulations was observed in females from the 14-day prior male exposure group (T_1_) (p = .04). Regarding NPD, the highest frequency of 11 to 15 empty days was observed in continuous mating females (T_0_; 26%), whereas the T_1_ group recorded the lowest percentage (9%; p = .03). Conversely, the highest frequency of prolonged empty days (more than 15 days) was recorded in the T_1_ group (46%) compared to the significantly lower and similar frequencies shared by T_0_ and T_2_ (p < .05).

**Table 2:** Mating indicators in 60-day-old nulliparous females.

	**T_0_ (Traditional)**	**T_1_ (Controlled 14-d)**	**T_2_ (Controlled 7-d)**	**p-value**
**Frequency of copulations at first mating, %**				
0 to 5 copulations	8	21	12	.07
6 to 10 copulations	58	37	67	.06
11 to 20 copulations	25 ^ab^	37 ^a^	17 ^b^	.04
More than 20 copulations	8	4	4	.39
**Non-productive days (from mating onset to conception), %**				
0 to 10 days	54	45	65	.29
11 to 15 days	26 *^b^*	9 *^a^*	17 *^ab^*	.03
More than 15 days	21 *^a^*	46 *^b^*	17 *^a^*	<.05

**Note:** T_0_: Traditional mating scheme (continuous cohabitation with the male); T_1_: Controlled mating with 14-day visual male exposure prior to mating; T_2_: Controlled mating with 7-day visual male exposure prior to mating. Value of *p* determined by Pearson's chi-square (χ2) test of proportions. *^a,b^* Different superscripts within the same row indicate statistically significant differences (p < .05). Values without superscripts indicate no significant difference (p > .05).

Gestation indicators and parturition timing for females that began mating at 60 days of age are presented in Table 3. The most frequent gestation length was concentrated between 65 and 68 days, showing no significant differences across the three treatments (p = .64). However, a significantly lower frequency of gestations lasting 69 to 70 days was recorded in females with 14-day prior exposure (T_1_; 13%), whereas T_0_ (27%) and T_2_ (34%) exhibited higher and statistically similar frequencies for this range (p = .02). Concurrently, T_1_ recorded a significantly higher percentage of prolonged gestations lasting 71 to 72 days (9%) compared to the other groups (p < .05). Parturition timing did not differ significantly among the evaluated mating systems (p > .05).

**Table 3:** Gestation indicators and parturition timing in nulliparous females.

	**T_0_ (Traditional)**	**T_1_ (Controlled 14-d)**	**T_2_ (Controlled 7-d)**	**p-value**
**Gestation length frequency, %**				
62 to 63 days	10 *^a^*	0 *^b^*	0 *^b^*	<.05
65 to 68 days	63	77	67	.64
69 to 70 days	27 *^a^*	13 *^b^*	34 *^a^*	.02
71 to 72 days	0 *^b^*	9 *^a^*	0 *^b^*	<.05
**Parturition timing, %**				
Late night/Early morning (0 to 6 h)	26	23	17	.45
Morning (6 to 12 h)	16	18	26	.32
Afternoon (12 to 18 h)	32	27	17	.15
Night (18 to 24 h)	26	32	39	.37

**Note:** T_0_: Traditional mating scheme (continuous cohabitation with the male); T_1_: Controlled mating with 14-day visual male exposure prior to mating; T_2_: Controlled mating with 7-day visual male exposure prior to mating. Value of *p* determined by Pearson's chi-square (χ2) test of proportions. *^a,b^* Different superscripts within the same row indicate statistically significant differences (p < .05). Values without superscripts indicate no significant difference (p > .05).

Finally, Table 4 summarizes the productive parameters and economic merit. Trends toward higher litter weight (p = .08) and a numerically higher number of live births were observed in females with 7-day prior male exposure (T_2_). Analysis of covariance (p > .05) showed no significant effect on productive parameters when using initial mating weight as a covariate.

**Table 4:** Productive parameters and economic merit according to the mating system.

	**T_0_ (Traditional)**	**T_1_ (Controlled 14-d)**	**T_2_ (Controlled 7-d)**	**p-value**
**Productive parameters**				
BW – mating onset, g	710 ± 58	634 ± 45	741 ± 38	.17
Liter size, n	3.4 ± 1.1	3.0 ± 0.8	3.6 ± 0.8	.08
Liter weight, g	488 ± 113	477 ± 102	544 ± 99	.23
Live births, n	2.8 ± 1.0	2.6 ± 1.1	3.2 ± 1.0	
**Economic merit**				
Gestation length, d	67.2	68.0	67.8	
Non-productive days, d	10.5 *^a^*	17.0 *^b^*	11.3 *^a^*	<.01
Feed intake, d	78.7	55.9	72.0	
Feed cost per female, PEN	10.05 *^a^*	7.23 *^c^*	9.27 *^b^*	<.01
Cost per live-born pup, PEN	3.54	2.74	2.92	

**Note:** T_0_: Traditional mating scheme; T_1_: Controlled mating with 14-day visual male exposure; T_2_: Controlled mating with 7-day visual male exposure. BW: Body Weight; PEN: Peruvian Sol (Local currency). Data for productive parameters is expressed as Mean ± Standard Deviation. The p-value for productive parameters was determined by ANOVA. p-values for non-productive days and costs were determined by Kruskal-Wallis. *^a,b,c^* Different superscripts within the same row indicate statistically significant differences (p < .05).

From an economic perspective, the feed cost per female was highest in the continuous mating system (T_0_) (p < .01), resulting in a higher production cost per live-born pup. In contrast, the 14-day prior exposure system (T_1_) showed the lowest feed consumption and cost per female. However, non-productive or empty days were significantly higher in the T_1_ group compared to the other treatments (p < .01).

## Discussions

Female puberty in guinea pigs typically occurs between 55 and 70 days of age (Matos et al., 2022), though this milestone exhibits high plasticity and is influenced by genetic, metabolic, and environmental factors (Steele et al., 2023). Notably, the 'male effect' can accelerate sexual maturity to as early as 26 days (Araníbar & Echevarría, 2014; Espinoza-Flores et al., 2020). In the present study, a 14-day visual exposure acted as a potent biosynchronizer, with 67% of females exhibiting estrus within the first 10 days of mating. This natural response matches the efficacy of pharmacological treatments, such as the 93% ovulation rate reported by Grégoire et al. (2012) using progestogens. Consequently, visual biostimulation offers a non-invasive, welfare-oriented alternative to optimize reproductive onset by leveraging the species' high sensory sensitivity.

A 14-day sensory pre-exposure to the male significantly accelerated puberty in nulliparous females, confirming early activation of the hypothalamus-pituitary-gonadal axis through the 'Vandenbergh effect' (Chasles et al., 2018). Mechanistically, priming pheromones and multimodal cues (visual and olfactory) upregulate GnRH pulsatility and LH secretion, modulating hormonal profiles and estrous behavior (McGlone et al., 2023). This biosynchronization not only induces timely estrus presentation but also optimizes reproductive fitness (Flanagan et al., 2011). Notably, in these precocial caviomorphs, this sociosexual priming is remarkably robust, validating visual exposure as an effective, non-invasive, and welfare-oriented alternative for guinea pig production systems (Trillmich et al., 2006).

In the present study, a total of 58% of females in the 14-day visual exposure group repeated estrus (46% twice and 12% three times), whereas the continuous mating and 7-day exposure groups showed lower recurrence rates (17% and 29%, respectively), leading to higher non-return rates (83% and 71%) and indicating greater fertilization success during the first estrus. These differences are hypothesized to be associated with male sexual satiety. In *Cavia porcellus*, most males reach satiety after a single ejaculation, losing immediate interest in the female (Young, 1969). The 14-day exposure group exhibited a higher copulatory frequency (37% of females copulated 11–20 times); given the short estrus duration in this species (8–11 h; Hargaden & Singer, 2012), this high mating frequency could potentially deplete sperm reserves. This is conceptually consistent with Rostellato et al. (2021), who reported a 12% and 32% reduction in sperm concentration by the second and third consecutive ejaculations. However, since seminal parameters and ejaculate quality were not directly evaluated, the proposed mechanisms of sperm depletion and acute sexual satiety must be interpreted as plausible biological hypotheses rather than confirmed causal factors. Nonetheless, this intense sexual activity in the 14-day group coincided with increased empty days (46% of females >15 d), supporting the hypothesis that excessive prior overstimulation might compromise male efficiency during actual mating by delaying timely conception (Lucy, 2019).

Gestation length in the vast majority of females ranged between 65 and 70 days across treatments, aligning with the species average of 68 days (Matos et al., 2022). However, a distinct dichotomy emerged: 10% of females in continuous mating (T_0_) showed shorter gestations (62–63 days), whereas females in the 14-day pre-exposure group (T_1_) exhibited a significant shift towards prolonged periods, with 9% of gestations extending to 71–72 days (p < .05). These extended gestations correlated with a trend toward smaller litter sizes, a phenomenon consistent with guinea pig reproductive physiology, where gestation length is inversely proportional to fetal number (Shomer et al., 2015). Although shorter gestations can compromise birth weight in other lagomorphs and rodents (Olateju & Chineke, 2022), prolonged gestation in small guinea pig litters typically allows for greater individual fetal development. Furthermore, our findings suggest that litter size was conditioned by copulatory dynamics; the high mating frequency triggered by a 14-day visual overstimulation likely induced male sexual satiety (Young, 1969). Given the limited ejaculate volume of *Cavia porcellus*, consecutive copulations could deplete sperm reserves and compromise the concentration required for successful multiple fertilizations (Hargaden & Singer, 2012).

Prolificacy is a cornerstone of productivity when integrated with efficient resource management (Labajova et al., 2016). Non-productive days are a critical profitability indicator, as their increase raises maintenance costs and reduces annual pup production per female (Pierozan et al., 2021). In this study, females under a 7-day visual biostimulation protocol showed NPD comparable to continuous mating, the conventional system in intensive guinea pig production (Shomer et al., 2015). Notably, the 7-day group recorded lower daily feed intake than the continuous system, optimizing costs by reducing feed expenditure per female and the production cost per live-born pup. Conversely, in the 14-day treatment, lower feed intake did not reflect efficiency but was linked to higher reproductive failures (increased NPD). Because voluntary feed intake naturally intensifies alongside fetal development, a higher proportion of open (non-pregnant) females inherently reduced the overall group intake average.

## Conclusions

In conclusion, a 7-day visual male exposure prior to mating represents a highly viable strategy to optimize reproductive management in intensive guinea pig production systems. This protocol effectively concentrates the first estrus presentation within a narrow window (predominantly between days 6 and 10), reduces repeated estrus rates compared to the 14-day overstimulation group, and minimizes the proportion of females with prolonged non-productive days. While key productive parameters such as litter size and litter weight do not differ significantly among treatments, the 7-day exposure protocol demonstrates superior economic and reproductive efficiency specifically regarding statistically supported variables, such as reduced female feed expenditure and minimized non-productive days.
