# ERRATUM: Effect of the duration of visual exposure to the male on the reproductive performance of nulliparous guinea pigs

**DOI:** 10.29374/2527-2179.bjvm013226

**Published:** 2026-09-03

Due to an authors honest mistake the article “Effect of the duration of visual exposure to the male on the reproductive performance of nulliparous guinea pigs” (https://doi.org/10.29374/2527-2179.bjvm003926), published in Brazilian Journal of Veterinary Medicine, 48, e003926, 2026, the affiliation numbers were incorrectly assigned to some authors.

On page 1, the authors’ names and affiliations should be corrected as follows:

**Where it reads:**

Hilario Noberto Pujada Abad^1^, Edinson Collas Ostos^1^, Roberto Carlos Tamayo Diaz^1^, Rufino Máximo Maguiña Maza^1^, Víctor Joselito Linares Cabrera^2^, Julio César Valencia Bardales^1^, Miguel Edmundo Lucho-Cerga^2^, Javier Hernán Luyo Flores^2^ & Felix Esteban Airahuacho-Bautista^2*^

^1^Universidad Nacional José Faustino Sánchez Carrión, Huacho, Lima, Peru.

^2^Instituto de Educación Superior Público Huando, Huaral, Lima, Peru.

**It should show:**

Hilario Noberto Pujada Abad^1^, Edinson Collas Ostos^1^, Roberto Carlos Tamayo Diaz^1^, Rufino Máximo Maguiña Maza^1^, Víctor Joselito Linares Cabrera^1^, Julio César Valencia Bardales^1^, Miguel Edmundo Lucho-Cerga^2^, Javier Hernán Luyo Flores^1^ & Felix Esteban Airahuacho-Bautista^1*^

^1^Universidad Nacional José Faustino Sánchez Carrión, Huacho, Lima, Peru.

^2^Instituto de Educação Superior Público Huando, Huaral, Lima, Peru.

The authors apologize for the errors.

